# Photosynthesis-Related Responses of Colombian Elite *Hevea brasiliensis* Genotypes under Different Environmental Variations: Implications for New Germplasm Selection in the Amazon

**DOI:** 10.3390/plants10112320

**Published:** 2021-10-28

**Authors:** Armando Sterling, Lised Guaca-Cruz, Edwin Andrés Clavijo-Arias, Natalia Rodríguez-Castillo, Juan Carlos Suárez

**Affiliations:** 1 Phytopathology Laboratory, Faculty of Basis Sciences, Sinchi Amazonian Institute of Scientific Research, Universidad de la Amazonía, Florencia 180001, Caquetá, Colombia; lisguacacruz@gmail.com (L.G.-C.); andresclavijoarias@gmail.com (E.A.C.-A.); narodriguezc16@gmail.com (N.R.-C.); 2 Natural Sciences and Sustainable Development, Faculty of Agricultural Sciences, Universidad de la Amazonía, Florencia 180001, Caquetá, Colombia; 3 Ecophysiology Laboratory, Agroecological Engineering Program, Faculty of Engineering, Universidad de la Amazonia, Florencia 180001, Caquetá, Colombia; ju.suarez@udla.edu.co

**Keywords:** rubber tree, elite genotypes, photosynthetic performance, leaf gas exchange, chlorophyll *a* fluorescence, leaf water potential, clonal selection

## Abstract

The objective of this study was to evaluate photosynthetic performance based on gas exchange traits, chlorophyll *a* fluorescence, and leaf water potential (*Ψ_L_*) in nine *Hevea brasiliensis* genotypes from the ECC-1 (Élite Caquetá Colombia) selection and the cultivar IAN 873 (control) in response to different climatic (semi-humid warm and humid warm climates), seasonal (dry and rainy periods), and hourly (3:00 to 18:00) variations that can generate stress in the early growth stage (two-year-old plants) in two large-scale clonal trials in the Colombian Amazon. The photosynthetic performance in 60% of the Colombian genotypes was slightly affected under the conditions with less water availability (dry period, semi-humid warm site, and between 9:00 and 15:00 h), as compared with IAN 873, whose affectation was moderate in terms of photosynthesis rates, but its water conservation strategy was strongly affected. The ECC 90, ECC 83, and ECC 73 genotypes had the best photosynthetic performance under conditions of greater water limitation, and ECC 35, and ECC 64 had a higher water status based on the leaf water potential, with intermediate photosynthetic performance. This germplasm has a high potential for selection in rubber tree breeding programs in future scenarios of climate change in the Colombian Amazon.

## 1. Introduction

The rubber tree (*Hevea brasiliensis* Willd. Ex A. Juss. Muell. Arg.) is a deciduous perennial species native to the Amazon rainforest and belongs to the Euphorbiaceae family [1]. Latex is extracted from this crop and is the main source of natural rubber for the tire industry, representing up to 90% of the rubber sold in international markets [2]. In Colombia, there are about 69,000 ha of rubber, with an estimated national production of 7500 tons per year, distributed mainly in the states of Meta, Santander, Antioquia, Vichada and Caquetá [3]. However, in regions with a high rubber tradition such as Caquetá (northwestern Amazon), cultivation has been promoted that has a reduced genetic base (four cultivars) that is highly susceptible to phytosanitary problems and has low physiological and productive performance [4,5,6]. This genetic base is represented in some 6700 ha, whose yield has decreased from 1.3 to 0.9 tons per ha per year in the last 15 years, affecting more than 1200 families that depend on this crop in the region [7]. The need to renew current plantations with new high-performance genetic materials is a priority in the Amazon region.

Consequently, genetic improvement programs aimed at obtaining new varieties of rubber with desirable genotypic and phenotypic characteristics, such as tolerance to abiotic and biotic stress, represent the main strategy for the long-term sustainability of this crop [8]. However, since rubber is a perennial crop, it requires more than six years to reach the productive phase and another five to six years to stabilize its productive cycle, so the process of selecting new genotypes in large-scale clonal trials can take 15 years or more before they can be released for commercial use [8,9]. Various early selection methods have been considered, including the use of physiological traits, to optimize evaluation criteria and reduce selection times [10].

Thus, one of the main approaches for early selection in juvenile plants for promising progeny uses physiological characteristics related to the efficiency of photosynthesis in various environments [11]. Traits such as gas exchange, pigment content (*Chl a, Chl b,* and carotenoids), chlorophyll a fluorescence, and water relations have shown high potential for the selection of desirable genotypes in various species [12,13,14,15]. The use of physiological traits, such as identification and selection criteria that improve the tolerance or sensitivity of the species to the stress factor, generates high heritability in the population of interest [10,16]. It has been reported, in tree species such as *Populus* [17], *Pinus* [18], *Eucaliptus* [14] and even in *Hevea* [13], that physiological traits are key to rapid selection in the first selection cycles.

Therefore, the new *Hevea* genotypes must have sufficient capacity to adapt to different stress conditions or unfavorable environments without affecting development during the growth phase and retaining the productive potential in the maturity phase [11,19].

Various studies carried out in the Colombian Amazon have sought to identify new introduced genotypes with desirable characters that are adaptable to different conditions and environments in the growth phase [13,20] and in the early production phase [21]. One of the greatest opportunities for the genetic improvement of *H. brasiliensis* lies in the regional elite germplasm [22]. We hypothesized that climatic, seasonal, or diurnal variations that can cause stress or limit photosynthetic performance in the growth phase of *H. brasiliensis* should have a lower impact on regional genotypes than on introduced traditional cultivars, which would help identify physiologically outstanding genotypes in the first selection cycles and potentially broaden the genetic base of this species in the region.

The objective of this study was to evaluate photosynthetic performance based on gas exchange traits, chlorophyll *a* fluorescence, and leaf water potential in nine *H. brasiliensis* genotypes from the ECC-1 (Élite Caquetá Colombia) selection and the cultivar IAN 873 (control) in response to different climatic, seasonal and hourly variations during the initial growth phase in large-scale clonal trials in the region, as an early selection strategy for new germplasm with desirable physiological characters with potential uses in the Colombian Amazon.

## 2. Results

### 2.1. Variations in the Microclimatic Parameters

**Figure 1:**
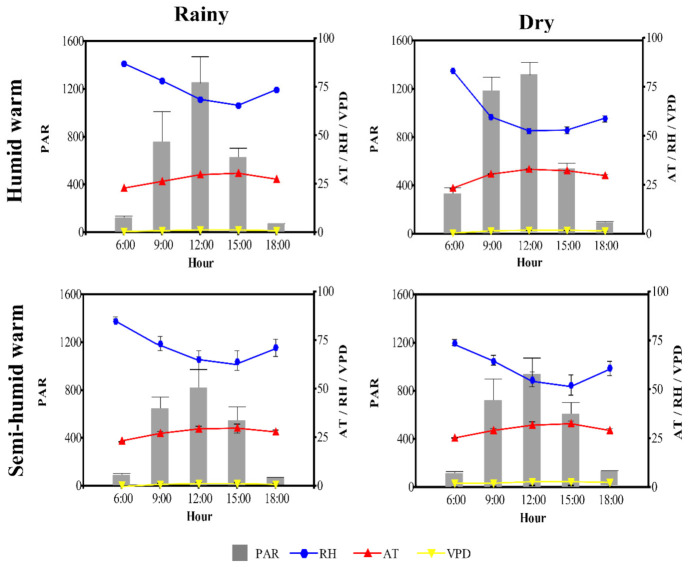
Diurnal microclimatic parameters for two sites with different climates: humid warm (El Paujil) and semi-humid warm (San Vicente del Caguán) in Caquetá (Northwestern Colombian Amazon) in two seasonal periods: rainy (April to June 2019) and dry (December 2019 to February 2020). Photosynthetically active radiation (*PAR*), relative humidity (*RH*), air temperature (*AT*) and vapor pressure deficit (*VPD*). The values represent the mean, and the bars the standard error, (*n* = 90).

In both sites, the highest means of *PAR*, *AT* and *VPD* were recorded in the dry period, while in the rainy period the highest mean *RH* was recorded (Figure 1). The highest *PAR* mean was recorded between 9:00 and 12:00 h during the dry (1179 to 1319.28 µmol photons m^−2^ s^−1^) and rainy periods (756.84 to 1249.33 µmol photons m^−2^ s^−1^) in the humid warm site. At 12:00 and 15:00 h, the highest *AT* mean (33.25 and 32.55 °C, respectively) was recorded during the dry period at the humid warm site, while the highest *VPD* mean (1.79 and 1.87 kPa) and the lowest mean relative humidity (60.65 and 53.75%) were recorded in the same hourly range during the dry period at the semi-humid warm site.

### 2.2. Changes in the Micro-Environmental Parameters at the Leaf and Soil Levels

On average, at the foliar level, the rainy period had the highest *RH* (71.61%), while the dry period had the highest *PAR* (513.37 µmol photons m^−2^ s^−1^), *VPD* (1.89 kPa) and *AT* (33.21 °C) values. In the dry period at the humid warm site, a maximum mean *PAR* of 1437.48 µmol photons m^−2^ s^−1^ (12:00 h), a maximum mean *AT* of 35.96 °C (12:00 h) and a maximum mean *VPD* of 2.58 kPa (18:00 h) were recorded. In the rainy period, the maximum mean *RH* was 73.60% (6:00 h). At the semi-humid site, the highest values of *PAR* (897.10 µmol photons m^−2^ s^−1^), *AT* (34.22 °C), *VPD* (1.99 kPa) and *RH* (71.94%) were recorded in the same periods and times as the humid warm site.

In the dry period at the semi-humid warm site, the most limiting hydric conditions were recorded in the soil in the hourly range between 9:00 and 15:00, with the lowest values of soil hydric potential (*Ψ_S_*) (−0.036 at −0.034 MPa) and volumetric water content (*VWC*) (34.05 to 35.35%), along with the highest soil temperature (*ST*) (27.81 to 28.17 °C) (Table S1; Supplementary Materials). Under the same seasonal and hourly conditions, the humid warm site had mean values of these parameters that varied between −0.011 and −0.020 MPa for *Ψ_S_*, 36.10 and 39.18% for *VWC*, and 27.51 and 27.85 °C for *ST*. In the rainy period, both sites had the lowest water limitations in the soil throughout the daily cycle, with values of *Ψ_S_*, *VWC* and *ST* that ranged between −0.011 and −0.009 MPa, 37.08 and 40.02%, and 26.52 and 27.39 °C, respectively.

### 2.3. Photosynthetic Responses to Light

Significant differences between genotypes were observed for the photosynthetic response curve to light (Table 1). The genotypes ECC 29, ECC 66, ECC 90, ECC 83 and ECC 25 presented the highest *A_max_* (>21 µmol CO_2_ m^−2^ s^−1^), while ECC 60 and ECC 35 had the lowest value, even lower than IAN 873 (control). However, this higher carbon fixation was not always related to a high *A_qe_* since the opposite occurred with ECC 29, which presented the lowest mean for this variable, while the ECC 60 genotype registered the highest average. The ECC 35 genotype had the highest *LCP* value, exceeding the remaining genotypes by more than 50%. *LSP* was above 1100 μmol photons m^−2^ s^−1^, where ECC 29 had the highest value, while ECC 60 had the lowest saturation point (900 μmol photons m^−2^ s^−1^). ECC 35 had the highest *R_d_* value, while ECC 73 and ECC 25 had more than a 60% reduction in substrate consumption.

### 2.4. Gas Exchange and Chlorophyll a Fluorescence

Highly significant variations were observed in the various gas exchange traits, chlorophyll *a* fluorescence and leaf water potential (*Ψ_L_*) (Table 2). There were very significant differences in the higher order interaction in 60% of the traits, with the exception of *Φ_PSII_*. In 35.71% of the cases, a very significant effect of the interaction between period, site and genotype was evidenced. Traits such as *A*, *WUEi*, *Ψ_L_* and various parameters of chlorophyll *a* fluorescence were not significant. In general, the interactions that combined spatial and temporal variations were more significant, and the fluorescence parameters of chlorophyll *a* were only slightly influenced.

The analysis of the main fixed effects on the variables (Table 3) showed that most of the gas exchange traits and *Ψ_L_* were significantly higher in the rainy period, while *WUEe, WUEi*, *LT*, *Φ_PSII_*, *ETR*, *F_v_/F_m_*, *F_v_’/F_m_´* were higher in the dry period.

At the humid warm site, the highest averages were recorded in most of the gas exchange and chlorophyll *a* fluorescence traits, while *LT*, *WUEe*, *WUEi*, and *Ψ_L_* were higher at the semi-humid warm site (Table 3). In general, between 9:00 and 12:00 h, the highest photosynthetic performance was evidenced with greater efficiency in the use of water, and lower *Ψ_L_* values. In contrast, between 3:00 and 6:00 h, higher *Ψ_L_* values were recorded. Between the genotypes, the greatest differences in some gas exchange traits were observed in the group with ECC 90 and ECC 73 and the group with ECC 64, ECC 25 and ECC 29 (Table 3). The chlorophyll *a* fluorescence parameters and *Ψ_L_* did not vary significantly in response to the only genotype effect, with the exception of *F_v_/F_m_* (Table 3). The ECC 73 genotype obtained the highest mean *F_v_/F_m_* (0.78), while ECC 35 presented the lowest value (0.75).

**Figure 2:**
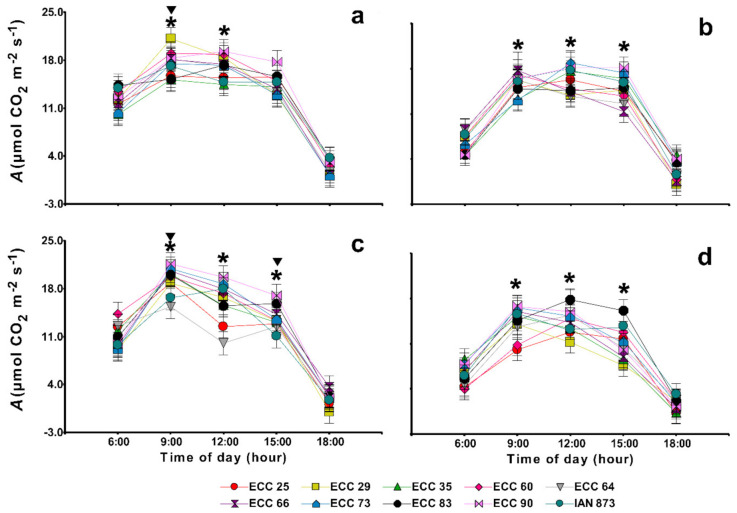
Diurnal net CO_2_ assimilation rate (*A*) (μmol CO_2_ m^−2^ s^−1^) at light saturation (1295 μmol photons m^−2^ s^−1^ and 90 s illumination time), in nine *Hevea brasiliensis* genotypes from the ECC-1 (Élite Caquetá Colombia) selection and the cultivar IAN 873 (control) in two seasonal periods at two sites with different climates in Caquetá (Northwestern Colombian Amazon). (**a**,**b**), humid warm site (El Paujil); (**c**,**d**), semi-humid site (San Vicente del Caguán); (**a**,**c**), rainy period; (**b**,**d**), dry period. In each site, means for the rainy and dry periods followed by an inverted triangle and for the genotypes followed by an asterisk (*) for each time of day were significantly different according to Fisher’s LSD test, (*p* < 0.05). Values represent the mean ± SE of four replications (*n* = 4).

However, the joint influence of climatic, seasonal, and hourly variations generated photosynthetic responses with significant differences in the mean photosynthetic rate (*A)* between the genotypes (Figure 2). Thus, in the rainy period (Figure 2a,c), the mean *A* reached maximum values at 9:00 h followed by a reduction at 12:00 h until reaching a minimum at 18:00 h at both sites, which contrasted with the dry period (Figure 2b,d) where maximum *A* values were observed at 12:00 h in 40% of the genotypes. Similarly, significant differences in the mean *A* were evidenced at 9:00 h between both periods at the humid warm (Figure 2a,b) and semi-humid warm (Figure 2c,d) sites. In addition, the same observation was made only at 15:00 h at the semi-humid warm site. In general, a greater variation in the mean *A* between genotypes was evidenced in the daily cycle of the semi-humid warm site in both periods (Figure 2c,d).

At the humid warm site, significant differences in the mean *A* between genotypes were found at 9:00 and 12:00 h in both periods (Figure 2a,b). In addition, significant differences were also evidenced at 15:00 h in the dry period (Figure 2b). The highest photosynthetic rate (21.15 µmol CO_2_ m^−2^ s^−1^) was observed in the ECC 29 genotype, as compared to the genotypes such as ECC 25 and ECC 83 (*p* < 0.05) at 9:00 h in the rainy period.

In the dry period, the variation between the genotypes was lower, and higher mean *A* values (18.18 to 19.18 µmol CO_2_ m^−2^s⁻ ^1^) were evidenced at 12:00 h in the ECC 90, ECC 73, ECC 35 and IAN 873 genotypes significantly higher than that evidenced in ECC 64, ECC 29, ECC 66 and ECC 83.

At the semi-humid warm site, significant differences in the mean *A* between the genotypes were found at 9:00, 12:00 and 15:00 h in both periods (Figure 2c,d). In the rainy period (Figure 2c), the ECC 90 and ECC 73 genotypes had higher photosynthetic rates (21.65 and 21.01 µmol CO_2_ m^−2^ s^−1^, respectively) at 9:00 h, which exceeded the photosynthesis observed in the IAN 873 and ECC 64 genotypes by more than 20%. At 9:00 in the dry period (Figure 2d), the ECC 90 and ECC 73 genotypes had higher mean *A* values that were similar to the values of IAN 873, while genotypes such as ECC 25 and ECC 60 significantly reduced their photosynthetic rates by more than 45%. In addition, the ECC 83, ECC 90, ECC 73 and ECC 64 genotypes reached higher rates (>16 µmol CO_2_ m^−2^ s^−1^) at 12:00 h, and ECC 83 had the highest mean *A* at 13:00 h, while ECC 29 was the least efficient in this time range.

**Figure 3:**
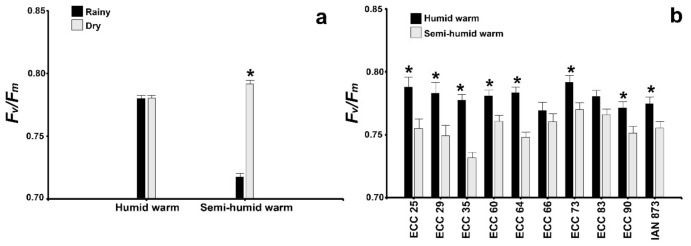
Maximum photochemical efficiency of PSII (*F_v_/F_m_*) for the significant interactions between site and period (**a**), and genotype with site (**b**). Means estimated for the rainy and dry periods for each site (humid warm and semi-humid warm) and for the sites followed by an asterisk (*) for each genotype were significantly different according to Fisher’s LSD test, (*p* < 0.05). Values represent the mean ± SE of four replications for the 10 genotypes (*n* = 40) (**a**), or of four replications for the two periods (*n* = 8) (**b**).

The maximum photochemical efficiency of PSII (*F_v_/F_m_*), based on the mean of the 10 genotypes, was significantly higher in the dry period (*F_v_/F_m_* = 0.79) as compared to the rainy period (*F_v_/F_m_* = 0.72) at the semi-humid warm site (Figure 3a). At the humid warm site, there was no significant variation in *F_v_/F_m_* between the periods. In addition, a significant influence of the genotype*site interaction was evidenced on *F_v_/F_m_* (Figure 3b). In 80% of the genotypes, significantly higher *F_v_/F_m_* averages were evidenced at the humid warm site, and the ECC 73 and ECC 25 genotypes had mean values (*F_v_/F_m_* = 0.79) higher than IAN 873 (*F_v_/F_m_* = 0.77). ECC 35 had the lowest mean *F_v_/F_m_* (0.73) at the semi-humid warm site. Other parameters, such as *F_v_’/F_m_’* and *ETR,* were similar to that observed in *F_v_/F_m_*. Thus, significant differences in the mean *F_v_’/F_m_’* were evidenced between the periods at the semi-humid warm site, with a maximum value of 0.76 in the dry period. *ETR* varied significantly between the periods at both sites, with maximum values of 102.35 and 86.31 in the dry period at the humid warm and semi-humid sites, respectively. *qP* was also similar to *F_v_/F_m_* in the genotype*site interaction. The genotypes ECC 64, ECC 90, ECC 73, ECC 60 and ECC 35 had significantly higher *qP* averages (0.91 to 0.95) at the humid warm site.

On the other hand, a significant effect of the site*period*hour interaction was evidenced on *ETR* and *Φ_PSII_*. The highest means of *ETR* (134.55 to 141.35) and *Φ_PSII_* (0.25 to 0.27) were evidenced between 9:00 and 15:00 in the dry period at the humid warm site.

### 2.5. Leaf Water Potential and Soil Water Status

**Figure 4:**
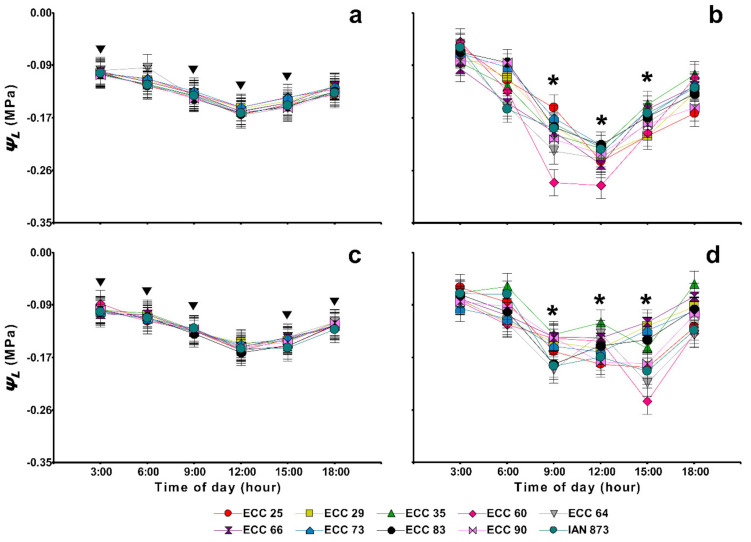
Diurnal leaf water potential (*Ψ_L_*) (MPa) in nine *Hevea brasiliensis* genotypes from the ECC-1 (Élite Caquetá Colombia) selection and the cultivar IAN 873 (control)in two seasonal periods at two sites with different climates in Caquetá (Northwestern Colombian Amazon). (**a**,**b**), humid warm site (El Paujil); (**c**,**d**), semi-humid site (San Vicente del Caguán); (**a**,**c**), rainy period; (**b**,**d**), dry period. In each site, means for the rainy and dry periods followed by an inverted triangle and for the genotypes followed by an asterisk (*) for each time of day were significantly different according to Fisher’s LSD test, (*p* < 0.05). Values represent the mean ± SE of four replications (*n* = 4).

At both sites and during the rainy period (Figure 4a,c), the leaf water potential (*Ψ_L_*) had a typical water behavior: *Ψ_L_* in the pre-dawn (3:00 h) decreased until reaching more negative values at 12:00 and 15:00 h, which began recovery around 18:00 h as the CO_2_ assimilation rate decreased at the end of the daily cycle (Figure 2a,c). However, in the dry period (Figure 4b,d), strong variations between genotypes were evidenced during the daily cycle at both sites, especially at 9:00 and 15:00 h at the humid warm and semi-humid warm sites, respectively. In addition, at 3:00, 9:00 and 15:00 h, significant differences were found between the two periods at both sites. Only the humid warm site had significant changes in the mean *Ψ_L_* between the two periods at 12:00 h (Figure 4a,b). Thus, at 3:00 h, the mean *Ψ_L_* was higher in the dry period at both sites (−0.08 to −0.07 MPa), as compared to the rainy period (−0.10 MPa). On the contrary, at 9:00 and 15:00, the water potentials in the rainy period were higher (−0.15 to −0.13 MPa) than that observed in the dry period (−0.20 to −0.17 MPa) at both sites.

Between the genotypes, minimal variations in the mean *Ψ_L_* were observed throughout the daily cycle in the rainy period at both sites (Figure 4a,c). However, strong significant variations were evidenced between 9:00 and 15:00 during the dry period at both sites (Figure 4b,d). In the dry period at the humid warm site (Figure 4b), genotype ECC 25 had higher leaf water potential (−0.16 MPa) at 9:00 h, as compared to genotypes such as ECC 60, ECC 64, ECC 90, ECC 66 and IAN 873 (all, <−0.18 MPa). At 12:00 h, genotype ECC 60 reached the lowest potential (−0.29 MPa) as compared to other genotypes (*p* < 0.05). In addition, genotypes ECC 29, ECC 25 and ECC 60 had a lower mean *Ψ_L_* at 15:00 h (all, −0.20 MPa).

On the other hand, in the dry period at the semi-humid warm site (Figure 4d), significant increases in the mean *Ψ_L_* were observed from 9:00 to 12:00 h for the genotypes ECC 35 and ECC 64 which had higher leaf water potentials (−0.11 and −0.12 MPa, respectively) at 12:00 h with less soil water availability (Table S1; Supplementary Materials). These mean values were significantly superior to that evidenced in IAN 873 (*p* < 0.05). In contrast, the two elite genotypes had lower mean *Ψ_L_* values at 15:00 h, similar to IAN 873 (all, *Ψ_L_* < −0.15 MPa).

### 2.6. Multidimensional Analysis of the Photosynthesis-Related Traits

**Figure 5:**
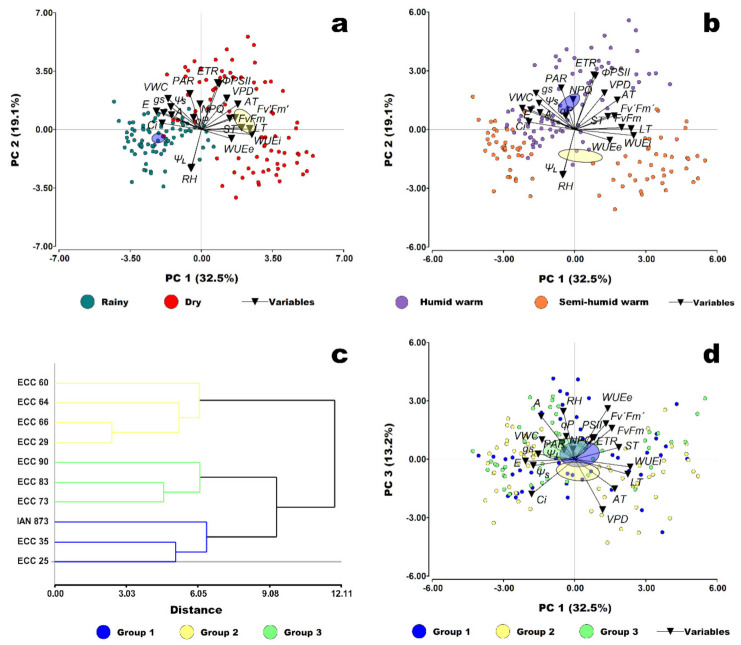
Multivariate plots of the studied variables (photosynthesis-related traits and micro-environmental parameters at the leaf and soil levels) in relation to the different analyzed effects. Biplot resulting from principal component analysis (PCA) of the variables and the observations grouped according to period (**a**) and site (**b**); (**c**) Dendrogram resulting from hierarchical clustering of nine *Hevea brasiliensis* genotypes from the ECC-1 (Élite Caquetá Colombia) selection and the cultivar IAN 873 (control); (**d**) PCA biplot of the variables and the observations grouped according to genotypes groups. 95% confidence ellipses around the centroid position. Net CO_2_ assimilation rate (*A*) (μmol CO_2_ m^−2^ s^−1^), transpiration rate (*E*) (mmol H_2_O m^−2^ s^−1^), stomatal conductance (*g_s_*) (mmol H_2_O m^−2^ s^−1^), intercellular CO_2_ concentration (*Ci*) (ppm), leaf temperature (*LT*) (°C), extrinsic water-use efficiency (*WUEe)* (μmol CO_2_ mmol H_2_O^−1^), intrinsic water-use efficiency (*WUEi)* (μmol CO_2_ mmol H_2_O^−1^), maximum photochemical efficiency of PSII (*F_v_/F_m_*), efficiency of excitation energy captured by open PSII reaction centers (*F_v_’/F_m_’*), photochemical quenching coefficient (*qP*), non-photochemical quenching coefficient (*NPQ*), current photochemical efficiency of PSII *(Φ_PSII_*), electron transport rate (*ETR*), leaf water potential (*Ψ_L_*) (MPa), soil water potential (*Ψ_S_*) (MPa), volumetric water content (*VWC*) (%), soil temperature (*ST*) (°C), photosynthetically active radiation (*PAR*) (μmol photons m^−2^ s^−1^), relative humidity (*RH*) (%), air temperature (*AT*) (°C) and vapor pressure deficit (*VPD*) (kPa).

The principal component analysis (PCA) synthesizes the characteristics related to photosynthesis, the microenvironmental parameters at the leaf and soil levels, and the main sources of variation in various ordination planes (Figure 5). Thus, the PCA captured 65% of the total variability with the first three principal components.

The gas exchange traits and soil water parameters were associated with the first principal component, which captured 32.5% of the total variability. The second component was represented by the chlorophyll *a* fluorescence parameters recorded in leaves not adapted to darkness and the foliar microenvironmental parameters, representing 19.1% of the captured information. Finally, the third component captured the information inherent to the fluorescence parameters of chlorophyll *a* in dark-adapted leaves and represented 13.2% of the total variability.

According to Figure 5a, the highest values of gas exchange parameters *A, E, g_s_*, and *Ci* were observed in the rainy period, along with the highest means of *Ψ_S_* and *VWC* in the soil microenvironment and a moderate increase in the relative humidity (*RH*) and the leaf water potential (*Ψ_L_*). On the contrary, there was a higher expression of the fluorescence parameters of chlorophyll *a* in the dry period under conditions with a higher *VPD* and higher leaf (*LT*), soil (*ST*), and air (*AT*) temperatures. Under these conditions, there was also a greater efficiency in the extrinsic and intrinsic use of water (*WUEe* and *WUEi*).

In general, the humid warm site had the highest values in the photosynthetic parameters related to gas exchange and chlorophyll *a* fluorescence, which in turn were associated with increases in *PAR, VPD, AT*, *Ψ_S_* and *VWC*, contrary to that observed at the semi-humid warm site, where there was a moderate increase in *Ψ_L_* and *RH* (Figure 5b), mainly favored during the rainy period (Figure 5a).

On the other hand, the hierarchical cluster analysis in Figure 5c identified three groups of genotypes, where IAN 873 (control) was associated with the ECC 35 and ECC 25 genotypes (group 1). The other genotypes were distributed in the two remaining groups.

According to Figure 5d and Table 3, group 1 in general, was associated with lower chlorophyll *a* fluorescence parameters values, and higher *WUEe* values under conditions with a higher *ST*. On the other hand, group 2 (ECC 29, ECC 60, ECC 64 and ECC 66) had a higher *WUEi*, *Ci* and *LT* in environments with a higher *AT* and *VPD*, and group 3 (ECC 90, ECC 83 and ECC 73) had genotypes with higher values of *A, E, g_s_* and *Ψ_L_* under conditions with a higher *PAR*, *Ψ_S_* and *VWC*. In addition, the IAN 873 and ECC 29 genotypes, in groups 1 and 2, respectively, were closer to group 3.

### 2.7. Girth Growth

Significant statistical differences were found between the 10 genotypes in each site after one year of growth (3-year-old trees) (Table 4). Significant differences were not evidenced between the sites. At the humid warm site, ECC 64, ECC 29, ECC 73, ECC 83 and ECC 90 had a higher mean girth than IAN 873 (*p* < 0.05), while at the semi-humid warm site, ECC 90, ECC 83 and ECC 73 had a greater vigor than IAN 873 (*p* < 0.05) (Table 4).

## 3. Discussion

The use of physiological indicators in plant breeding programs is essential for optimizing and reducing selection cycles, which have been mainly based on growth and productivity indicators [23,24,25], obviating important photosynthetic performance-related traits from an ecophysiological approach, since, in most cases, the genotypes selected with classical agronomic criteria are not tolerant to adverse environmental conditions derived from climate variability; nor will they be tolerant under the future climate change scenarios projected for the Amazon region [26].

Thus, various physiological traits related to the photosynthetic performance of *H. brasiliensis*, such as the photosynthetic rate, water use efficiency, girth growth, latex yield, and biomass production are influenced by the interaction of the genotype with environmental variations that allow the species to adapt or acclimate to limiting or stressful conditions [13,19,20,25,27,28].

In this study, under water deficits conditions (dry period), especially between 9:00 and 15:00 h, the greatest increase in environmental variables such as *AT, PAR*, *VPD* and *ST*, as well as a decrease in *RH*, *VWC* and *Ψ_S_*, generated a significant reduction in the photosynthetic rates and promoted other adaptation mechanisms related to an increase in the efficient use of water (*WUEe* and *WUEi*)*,* the pre-dawn increase in the leaf water potential (*Ψ_L_*), and the optimization of processes related to the photochemical metabolism (*ΦPSII, ETR, F_v_/F_m_* and *F_v_’/F_m_’*). In addition, in less humid environments (semi-humid warm climate), the strategy was mainly to conserve the water resource by increasing the efficient use of water and maintaining higher levels of early *Ψ_L_* in the morning (3:00 to 6:00 h) and at the end of the afternoon (18:00 h). However, some genotypes increased their photosynthetic rates towards noon (12:00 h) by increasing their leaf water potential and the efficient use of water between 9:00 and 12:00 h. On the contrary, during the rainy period and in more humid environments, the increase in soil water availability (*Ψ_S_* and *VWC*) and the decrease in *VPD* favored an increase in the mean values of various traits associated with photosynthetic performance, such as *A, E, g_s_, Ci, qP,* and *Ψ_L_*. When the water supply is sufficient, more carbon is transported to the roots, increasing the absorption and transport capacity of water per unit of leaf area, favoring the capacity to regulate the water-carbon exchange and photosynthesis [29,30].

The limitations evidenced in the gas exchange parameters during the dry period, which were associated with an increase in *VPD*, reflect the need to conserve water as a strategy to survive droughts [20,21], which, in this study at the time of highest photosynthetic efficiency (9:00 h), caused reductions between 45 and 47% in photosynthetic rates in some genotypes such as ECC 25 and ECC 60 and reductions between 31 and 35% in leaf water potentials in others such as IAN 873 and ECC 64, with respect to the rainy period (Figure 2 and Figure 3).

Increasing the efficient use of water, regulating transpiration and stomatal conductance as a direct response to water availability during the middle of the day are the main strategies used by plants to maintain high photosynthetic rates [31,32,33,34,35,36]. In this study, in the dry period at the semi-humid warm site, the genotypes ECC 64 and ECC 83, increased their photosynthetic rates (Figure 2d) and leaf water potentials (Figure 4d) between 9:00 and 12:00 h. In particular, at 12:00 h, ECC 83 had a mean *A* significantly higher than IAN 873 (*p* < 0.05), and ECC 64 had a mean *Ψ_L_* higher than IAN 873 (*p* < 0.05). This may be related to a decrease in hydraulic conductivity as the result of an increase in tension and cavitation of the xylem, minimizing the leaf water imbalance [37,38]. High *VPD* values associated with a higher volumetric content of water in the soil at noon favor the efficiency of daily transpiration, which reduces the loss of foliar water [39,40].

On the contrary, in the dry period at the humid warm site, other genotypes, such as ECC 90 and ECC 73, increased their photosynthetic rates at 12:00 h but decreased their leaf water potentials as a result of a higher stomatal conductance (891.23 and 774.48 mmol H_2_O m^−2^ s^−1^, respectively) and a higher transpiration rate (5.30 and 5.05 mmol H_2_O m^−2^ s^−1^, respectively).

Similarly, increases in *VPD* and *AT* that directly affect the photosynthetic rate by reducing the supply of CO_2_ to rubisco as a result of a decrease in *g_s_* and *Ci* in the dry period [41,42], are more evident in young plants than adults since they do not have a sufficient volume of roots that would facilitate better use of the water available in deep levels of the soil [43]. However, in this study, the young individuals (two years old) of the Colombian genotypes had more efficient use of water and avoided important reductions in the leaf water potential during the dry period between 9:00 and 12:00 h, as compared with some 9-year-old introduced genotypes evaluated in the same environments, which had reductions in *Ψ_L_* above 50% [20], and decreases in the photosynthetic rate, between 30 and 40% [13] as compared to Colombian genotypes.

In the rainy period, the stomatal conductance was high in most genotypes, allowing a greater fixation of CO_2_ that translated into a maximum use of soil water for transpiration, which was expressed by a lower *WUE*, results that coincide with that reported by Blum et al. [44], but contradict other studies that have indicated greater photosynthesis in the dry period of the Amazon region [13,45,46]. This reflects contrasting photosynthetic responses at the intra-specific level of *H. brasiliensis*, in which some genotypes require greater water availability to maintain high photosynthetic rates; others are more efficient at using water to maintain these rates, and others are affected in terms of their efficiencies, photosynthesis and water status under drought conditions [11,19,20]. In this study, in both environments (semi-humid warm and humid warm), the dry period and range between 9:00 and 12:00 h saw 60–70% of the genotypes with minimal reductions (<30%) in photosynthetic rates while others (30–40%) had moderate increases in photosynthesis (>20%). These results showed the high photosynthetic performance of Colombian genotypes, which reflected a greater ability to adapt to limiting conditions or drought stress in less humid environments and during the dry period [45]. These genotypes also showed relatively high transpiration and stomatal conductance rates, comparable to those reported for adult *H. brasiliensis* trees [13,46].

When analyzing the leaf temperature in relation to the carbon assimilation rate, the pre-dawn (3:00 h) and noon (12:00 h) efficient use of water and leaf water potentials in the Colombian genotypes had a partial isohydric behavior in the dry period [47], similar to that reported by Sterling et al. [13] in 9-year-old introduced genotypes. Thus, the most photosynthetically efficient genotypes (Figure 3) and vigorous (ECC 90, ECC 83 and ECC 73) (Table 4), kept the leaf temperature lower in relation to that of the air, which allowed them to maintain the assimilation of CO_2_ without impacts from high temperatures [48], and exchanged water for carbon and biomass [49,50]. Consequently, when the increase in CO_2_ occurred, the relationship between carboxylation and oxygenation of Rubisco increased, translating into greater net photosynthesis and a reduction in photoreduction [51]. In addition, these genotypes also showed high efficiency in quantum conversion (*A_qe_*) and lower light compensation points (*PCL*) (Table 1).

In the dry period, the rubber trees had a high photochemical efficiency (higher *Φ_PSII_, ETR, F_v_/F_m_* and *F_v_’/F_m_’* values) under water limiting conditions (Table 3), response pattern that was more evident for *F_v_/F_m_* and *F_v_’/F_m_’* in semi-humid warm site (Figure 3a) and Colombian genotypes (Figure 3b). This response could be due to partial isohydric behavior presented during the dry period (Figure 4) may have protected the photochemical apparatus from excess light energy under the conditions of drought stress [51], and prevented photodamage in the compounds of photosystem II [52,53,54,55]. These results are similar to that evidenced by Rodrigues et al. [53] in savanna plants that had a higher photochemical efficiency during the peak of the dry season, compared to beginning of the wet season and peak of the wet season. A similar pattern was reported by Sterling et al. [13] in introduced *H. brasiliensis* genotypes in the dry period at humid warm sites in Colombian Amazon.

Finally, the low *NPQ* values evidenced in this study were very similar to Sterling et al. [13] under similar environmental conditions. This indicates that, in our study, there was also no photoinhibition from excess light or water deficits, which means that the rubber trees did not use the heat dissipation pathway to protect the photosynthetic apparatus from the zeaxanthin cycle [56]. On the contrary, in our study, photochemical dissipation was more evident with higher *qP* values than in other reported for rubber trees under water stress conditions [43] or biotic stress [57], in which there were higher *NPQ* values in response to these types of stress.

## 4. Materials and Methods

### 4.1. Study Area

This study was carried out at two sites with different tropical climates, located in a hilly landscape (undulating relief with slopes no greater than 25%) in the department of Caquetá (northwestern Colombian Amazon): El Paujil, in the Moravia rural settlement area (1°31′38.46″ north and 75°17′32.59″ west, at 282 m above sea level), and San Vicente del Caguán, in the Buenos Aires settlement area (2°2′40.8″ north and 74°55′11.7″ west, at 346 m above sea level). According to the Caldas-Lang climate classification [58], San Vicente del Caguán has a semi-humid warm climate (mean temperature of 25.4 °C, mean relative humidity of 79%, precipitation of 2503 mm year^−1^ and a Lang index of 98.6), and El Paujil has a humid warm climate (mean temperature of 25.8 °C, mean relative humidity of 81.2%, precipitation of 3490 mm year^−1^ and a Lang index of 135.3).

The Caquetá region has a monomodal precipitation regime [59] with two marked seasonal periods: dry period (November to February) and rainy period (March to June). The other months correspond to a transition between the rainy and dry periods.

The Caquetá soils are mainly clayey, with ferric aluminum oxides, high acidity, high aluminum contents, and very low fertility [58]. The soils in El Paujil have a pH of 4.91 (extremely acidic), an electrical conductivity of 0.07 dS m^−1^, a cation exchange capacity of 11.16 meq 100g^−1^, an organic matter content of 2.23%, a clay texture (52.75% clay, 21.13% sand and 26.13% silt), a total nitrogen content of 0.11%, a mean saturation of 27.86% and an aluminum saturation of 83.7%. In San Vicente del Caguán, the pH is 4.85 (extremely acidic), with an electrical conductivity of 0.06 dS m^−1^, a cation exchange capacity of 10.31 meq 100g^−1^, an organic matter content of 1.69%, a clay texture (55.25% clay, 32.50% sand and 12.25% silt), a total nitrogen content of 0.08%, a mean saturation of 30.88% and an aluminum saturation of 66.9%.

In the study area, the microclimatic parameters: photosynthetically active radiation (*PAR*) (μmol photons m^−2^ s^−1^), relative humidity (*RH*) (%), air temperature (*AT*) (°C) and vapor pressure deficit (*VPD*) (kPa) were provided by an Automatic Weather Portable Station (Decagon Devices Inc, Pullman, WA, USA), and the data were calculated in two periods: dry (December 2018 to February 2019), and rainy (April to June 2019) with average hourly data at 3:00, 6:00, 9:00, 12:00 and 18:00, following the methodology used by Sterling et al. [13,20].

### 4.2. Plant Material

The plant material was young, 2-year-old trees in growth stage 3 and code 33 (BBCH scale) (BBCH scale) [60] (i.e., in the pre-tapping phase), using nine *H. brasiliensis* genotypes from the ECC-1 (Élite Caquetá Colombia) selection: ECC 25, ECC 29, ECC 35, ECC 60, ECC 64, ECC 66, ECC 73, ECC 83, and ECC 90 [22,61], and a widely distributed, introduced cultivar in Colombia, the IAN 873 clone (control) [62]. The Colombian genotypes came from unknown parents (natural cross-pollination) and were obtained for the first time in 2009 with asexual propagation (cloning) using elite rubber trees with a sexual origin from producer farms in Caquetá [22]. These materials were initially described and characterized with small-scale trials using morphoagronomic and molecular descriptors [22,63,64,65,66], and subsequently reported in various varietal selection studies that used large-scale clonal trials in Caquetá [63,64,65]. The nine genotypes from the ECC-1 selection were chosen from an elite germplasm collection (99 genotypes) because they had the best performance in growth, early yield tests and resistance to diseases observed in a small-scale trial in Caquetá [22]. The IAN 873 cultivar was chosen as the control because it is one of the most widely planted in countries, such as Colombia but its yield has decreased by more than 30% over the last 15 years [7].

### 4.3. Experiment Design and Field Trial Maintenance

In April of 2017, an experiment known as Large-Scale Clonal Trial (LSCT) [66] was established at each study site, as done in previous studies [13,20,21,67]. Thus, each LSCT was conducted with a randomized complete block design with 10 treatments (genotypes) and four replications (plots) randomly arranged in Fisher blocks, with 60 trees per genotype and per replication. The planting distance was 7.0 m × 3.0 m, providing a density of 476 trees ha^−1^ for a total area of 5.04 ha. The area of one plot was 1260 m^2^ and corresponded to 60 trees organized in 3 rows of 20 trees.

Each LSCT had fertilization management every six months with applications of organic matter (476 kg ha^−1^ year^−1^) and a mixture of NPK fertilizer (NH_4_^+^ 15%, P_2_O_5_ 15%, and K_2_O 15%) with minor elements (CaO 18%, MgO 6%, S 1.6%, B 1%, Cu 0.14%, Mo 0.005%, and Zn 2.5%) at a dose of 107 kg ha^−1^ year^−1^, similar to that used in previous studies [13,20]. Mechanical weed controls were performed every three months. Phytosanitary controls were not carried out.

### 4.4. Photosynthesis-Related Traits

#### 4.4.1. Photosynthetic and Micro-Environmental Traits at the Leaf Level

Variables related to photosynthetic response curves to light (*A/PAR*), gas exchange, foliar microenvironment and chlorophyll a fluorescence were obtained according to the methodology of Sterling et al. [13,20]. These measurements were performed using a portable photosynthesis system (CIRAS-3 PP Systems, Amesbury, MA, USA) coupled to chlorophyll *a* fluorescence module (CFM-3 PP Systems, Amesbury, MA, USA), maintaining a CO_2_ flow at a concentration of 390 μmol mol^−1^, a cuvette temperature (*TC*) of 27 °C, a mean relative humidity (*RH*) of 70% and a mean vapor pressure deficit (*VPD*) of 2.5 kPa.

The *A/PAR* curves determined the constant saturating *PAR* (1295 μmol photons m^−2^ s^−1^), which was used in all foliar photosynthetic and micro-environmental measurements in all genotypes. The *PAR* intensity was modulated in decreasing order at 16 levels between 2500 and 0 μmol photons m^−2^ s^−1^, between 9:00 and 12:00 h (hourly range of maximum photosynthesis) [13]. The variables maximum net CO_2_ assimilation rate (*A*_max_) (µmol CO_2_ m^−2^ s^−1^), light compensation point (*LCP*) (μmol photons m^−2^ s^−1^), light saturation point (*LSP*) (μmol photons m^−2^ s^−1^), dark respiration rate (*R_d_*) (µmol CO_2_ m^−2^ s^−1^) and apparent quantum efficiency (*A_qe_*) (µmol CO_2_ µmol photons-^1^) were estimated for each genotype adjusting the Mitscherlich model [13,67].

Measurements of the gas exchange, foliar microenvironment and chlorophyll *a* fluorescence were taken at each site, defined according to the climatic classification (humid warm and semi-humid warm) in each period (dry and rainy) and in a diurnal cycle every 3 h (6:00, 9:00, 12:00, 15:00 and 18:00) on sunny days on two healthy leaves with stage D physiological maturity [68], in the middle third of the canopy from four trees per replication of each genotype [13,20]. Then, for each variable the average of the four trees per replication of each genotype was used for the data analysis [20].

The gas exchange traits were: net CO_2_ assimilation rate (*A*) (μmol CO_2_ m^−2^ s^−1^), transpiration rate (*E*) (mmol H_2_O m^−2^ s^−1^), stomatal conductance (*g_s_*) (mmol H_2_O m^−2^ s^−1^), intercellular CO_2_ concentration (*Ci*) (ppm), leaf temperature (*LT*) (°C), extrinsic water-use efficiency (*WUEe* = *A*/*E*) and intrinsic water-use efficiency (*WUEi = A*/*g_s_*), while, the foliar micro-environmental parameters were: *PAR*, *RH*, *VPD* and *AT* [13,20].

The chlorophyll *a* fluorescence variables maximum photochemical efficiency of PSII (*F_v_/F_m_*), efficiency of excitation energy captured by open PSII reaction centers (*F_v_’/F_m_’*), photochemical quenching coefficient (*qP*) and non-photochemical quenching coefficient (*NPQ*) were simultaneously measured in the pre-dawn (3:00 h) in the same leaves used for the gas exchange variables, while the variables current photochemical efficiency of PSII *(Φ_PSII_*) and electron transport rate (*ETR*) were measured in leaves adapted to light in the diurnal cycle [13,69,70,71,72,73,74,75,76,77,78].

#### 4.4.2. Leaf Water Potential and Soil Water Status-Related Traits

In the same trees and under the same experiment and environmental conditions where the gas exchange variables, foliar microenvironment and chlorophyll *a* fluorescence were evaluated, the variables related to the water status of the soil-plant system were measured in a daily cycle every 3 h (3:00, 6:00, 9:00, 12:00, 15:00 and 18:00) following the methodology of Sterling et al. [20]. Thus, the leaf water potential (*Ψ_L_*) (MPa) was measured on four different leaves with stage D from four trees per replication of each genotype using a Schöllander pressure chamber (PMS Model 1515D, USA). The mean value of the four trees per replication of each genotype was used for the data analysis. In the soil, the following parameters were estimated: soil water potential (*Ψ_S_*) (MPa) using MPS-6 sensors MPS-6 (Decagon Devices Inc, Pullman, WA, USA), and volumetric water content (*VWC*) (%) and soil temperature (*ST*) (°C) using EC-5 sensors (Decagon Devices Inc., Pullman, WA, USA); both sensors were located at a depth of 40 cm and one meter away from the tree. A ProCheck PC4254 Datalogger (Decagon Devices Inc, Pullman, WA, USA) was used for data logging and storage.

#### 4.4.3. Assessment of Girth Growth

The girth (cm) growth in 3-year-old trees for the 10 *H. brasiliensis* genotypes was evaluated at the two sites (i.e., after 1 year of growth in the LSCT) to analyze vigor [25], and support the photosynthesis-related traits.

### 4.5. Data Analysis

Linear mixed-effects (LME) models for longitudinal data [69] were used to analyze the traits of leaf gas exchange, chlorophyll *a* fluorescence and leaf water potential. The site (humid warm and semi-humid warm), period (dry and rainy), genotype (nine Élite Caquetá Colombia and IAN 873), hour (6:00 to 18:00 h or 3:00 to 18:00 h) and their interactions were included as fixed effects, while the blocks nested at the sites and the plots associated to the genotypes within the blocks were included as random effects [13]. The assumptions of normality and homogeneity of variances were validated by exploratory residual analysis, and the residual correlation was considered to analyze the repeated measurements over time. The separation of means was performed using Fisher’s LSD test with a significance level of 5%. A hierarchical cluster analysis based on Euclidean distance and Ward’s method was used to group the genotypes based on photosynthesis-related traits. A principal component analysis (PCA) was used to study the relationships between the fixed effects and photosynthesis-related traits. Finally, a LME model with analysis of covariance (covariate: girth in 2-year-old trees) was used to analyze growth (girth in 3-year-old trees) and back up the physiological measurements. The LME models were fitted with the *lme* function in the R package nlme [70] in R language v. 4.0.3 [71], and the interface in InfoStat v. 2020 [72]. The PCA and hierarchical clustering were performed with InfoStat v. 2020.

## 5. Conclusions

The present study showed how climatic, seasonal, and diurnal variations can affect the photosynthetic performance of *H. brasiliensis* during the early growth stage, and how genotypic variation can be used for the early selection of genotypes with desirable photosynthetic characteristics. This study, and others like it, are essential to plant breeding to optimize the selection criteria and reduce the breeding cycle towards, obtaining new plant varieties that are not only more productive but also more tolerant of different stress factors that can limit or drastically affect the physiological plasticity of plants. Therefore, their adaptive strategies for survival and development under future climate change scenarios will improve.

In our study, the photosynthetic performance in 60% of the Colombian elite *H. brasiliensis* genotypes was little affected under the conditions of less water availability (dry period, semi-humid warm site, and time range between 9:00 and 15:00 h), as compared with cultivar IAN 873 (control), which was moderately affected in terms of photosynthesis rates but strongly affected in relation to its water conservation strategy. Greater increases in water potential in the pre-dawn (3:00 h) or in the efficiency to capture, use and dissipation of light energy in photosystem II during the dry period for increasing the photosynthetic rate, and the leaf water potential between 9:00 and 12:00 h were the main strategies used by the Colombian genotypes to minimize the impacts of lower water availability in the soil during that period.

In conclusion, ECC 90, ECC 83, and ECC 73 were the Colombian genotypes with the best photosynthetic performance and girth growth under conditions of greater water limitation, and ECC 35 and ECC 64 had a higher water status based on leaf water potential, with intermediate photosynthetic performance under the same conditions. This indicated that this germplasm has a high potential for selection in programs for the improvement of *H. brasiliensis* to broaden the genetic base of this species in the region using regional genotypes with high photosynthetic performance and tolerance to water deficits in future scenarios of climate change in the Colombian Amazon. The use of these genotypes in the Amazon will imply a strategy based on the selection of physiologically desirable genotypes, but also desirable in terms of tolerance to pests and diseases, yield, and latex quality. Thus, the final stage will involve analyzing the physiological, agronomic, and phytosanitary parameters associated with the productive performance (yield and latex-related traits) in these genotypes in the post-tapping phase in large-scale clonal trials before making a final recommendation for small commercial scale producers.

## Figures and Tables

**Table 1:** Traits derived from the photosynthetic light-response curves (*A/PAR*) in nine *Hevea brasiliensis* genotypes from the ECC-1 (Élite Caquetá Colombia) selection and the cultivar IAN 873 (control) in Caquetá (Northwestern Colombian Amazon). Maximum net CO_2_ assimilation rate (*A_max_*) (µmol CO_2_ m^−2^ s^−1^), light compensation point (*LCP*) (μmol photons m^−2^ s^−1^), light saturation point (*LSP*) (μmol photons m^−2^ s^−1^), dark respiration rate (*R_d_*) (µmol CO_2_ m^−2^ s^−1^) and apparent quantum efficiency (*A_qe_*) (µmol CO_2_ µmol photons^−1^).

Genotype	*A_max_*	*LCP*	*LSP*	*R_d_*	*A_qe_* (×10^−3^)
ECC 25	21.30 ± 0.29 ^d^	47.80 ± 3.29 ^c^	1486.92 ± 16.69 ^b^	−1.69 ± 0.13 ^a^	1.6 ± 0.1 ^e^
ECC 29	24.83 ± 0.43 ^a^	45.64 ± 4.14 ^c^	1580.70 ± 20.70 ^a^	−1.80 ± 0.19 ^b^	1.5 ± 0.1 ^e^
ECC 35	16.79 ± 0.28 ^g^	126.73 ± 3.89 ^a^	1338.62 ± 22.85 ^c^	−4.57 ± 0.16 ^d^	1.9 ± 0.1 ^c^
ECC 60	15.25 ± 0.28 ^h^	48.03 ± 4.78 ^c^	900.84 ± 27.33 ^e^	−2.11 ± 0.26 ^c^	2.7 ± 0.2 ^a^
ECC 64	20.51 ± 0.21 ^e^	50.89 ± 2.50 ^c^	1262.78 ± 13.23 ^c^	−2.01 ± 0.12 ^b^	1.9 ± 0.1 ^c^
ECC 66	22.28 ± 0.29 ^b^	61.50 ± 2.99 ^b^	1500.62 ± 15.89 ^a^	−2.20 ± 0.13 ^c^	1.6 ± 0.1 ^e^
ECC 73	18.59 ± 0.60 ^f^	41.32 ± 8.49 ^d^	1192.61 ± 44.35 ^d^	−1.58 ± 0.38 ^a^	2.0 ± 0.2 ^b^
ECC 83	21.71 ± 0.21 ^c^	48.42 ± 2.35 ^c^	1402.88 ± 12.08 ^b^	−1.82 ± 0.10 ^b^	1.7 ± 0.0 ^d^
ECC 90	21.80 ± 0.36 ^c^	45.32 ± 4.28 ^c^	1257.21 ± 22.28 ^d^	−1.96 ± 0.22 ^b^	1.9 ± 0.1 ^c^
IAN 873	20.44 ± 0.25 ^e^	58.77 ± 3.39 ^b^	1210.06 ± 18.81 ^d^	−2.30 ± 0.16 ^c^	2.0 ± 0.1 ^b^

Values represent the mean ± SE of four replications (*n* = 4). Means in each column followed by the same letter do not differ statistically (Fisher’s LSD test, *p* < 0.05).

**Table 2:** Analysis of variance of the fixed effects on the photosynthesis-related traits at the leaf level in nine *Hevea brasiliensis* genotypes from the ECC-1 (Élite Caquetá Colombia) selection and the cultivar IAN 873 (control) on a diurnal cycle in two seasonal periods at two sites with different climates in Caquetá (Northwestern Colombian Amazon). Period (P), site (S), genotype (G), hour (H), and their interactions on net CO_2_ assimilation rate (*A*) (μmol CO_2_ m^−2^ s^−1^), transpiration rate (*E*) (mmol H_2_O m^−2^ s^−1^), stomatal conductance (*g_s_*) (mmol H_2_O m^−2^ s^−1^), intercellular CO_2_ concentration (*Ci*) (ppm), leaf temperature (*LT*) (°C), extrinsic water use efficiency (*WUEe*) (μmol CO_2_ mmol H_2_O^−1^), intrinsic water use efficiency (*WUEi*) (μmol CO_2_ mmol H_2_O^−1^), actual photochemical efficiency of PSII (*Φ_PSII_*), electron transport rate (*ETR*), maximum photochemical efficiency of PSII (*F_v_/F_m_*), efficiency of excitation energy captured by open PSII reaction centers (*F_v_’/F_m_’*), photochemical quenching coefficient (*qP*), non-photochemical quenching coefficient (*NPQ*) and leaf water potential (*Ψ_L_*) (MPa).

Variables	*F* Based *p* Values														
	P	S	G	H	P × S	P × G	P × H	S × G	S × H	G × H	P × S × G	P × S × H	P × G × H	S × G × H	P × S × G × H
*A*	<0.0001	0.0146	0.0107	<0.0001	0.0572	0.3056	<0.0001	0.6770	0.0011	0.1680	0.8396	0.0012	0.0349	0.0849	0.0005
*E*	<0.0001	0.0032	<0.0001	<0.0001	<0.0001	<0.0001	0.3156	0.0079	<0.0001	0.0045	<0.0001	<0.0001	0.0556	0.0245	0.0179
*g_s_*	<0.0001	0.0006	<0.0001	<0.0001	<0.0001	<0.0001	<0.0001	0.8721	<0.0001	0.0003	<0.0001	<0.0001	<0.0001	<0.0001	<0.0001
*Ci*	<0.0001	0.0939	0.0003	<0.0001	<0.0001	0.0002	<0.0001	0.0758	<0.0001	0.4206	<0.0001	0.0002	<0.0001	0.0793	<0.0001
*LT*	<0.0001	0.0166	<0.0001	<0.0001	<0.0001	<0.0001	<0.0001	<0.0001	<0.0001	<0.0001	<0.0001	<0.0001	<0.0001	<0.0001	<0.0001
*WUEe*	0.0011	0.0690	<0.0001	<0.0001	<0.0001	0.1040	<0.0001	0.0375	<0.0001	0.0036	<0.0001	<0.0001	0.0399	0.0345	<0.0001
*WUEi*	<0.0001	0.0223	0.9444	<0.0001	0.0001	0.9815	<0.0001	0.9368	<0.0001	0.0096	0.9566	<0.0001	0.0001	0.0159	0.0018
*Φ_PSII_*	<0.0001	0.0066	0.0889	<0.0001	0.0611	0.0289	<0.0001	0.7566	<0.0001	0.0767	0.0253	<0.0001	0.0216	0.1567	0.0737
*ETR*	<0.0001	0.0062	0.0583	<0.0001	0.0283	0.0259	<0.0001	0.6949	<0.0001	0.1168	0.0367	<0.0001	0.0031	0.1494	0.0148
*F_v_/F_m_*	<0.0001	0.0001	0.0010	-	<0.0001	0.1665	-	0.0269	-	-	0.6266	-	-	-	-
*F_v_´/F_m_´*	<0.0001	0.0151	0.4053	-	<0.0001	0.4018	-	0.2829	-	-	0.5865	-	-	-	-
*qP*	0.0174	0.0034	0.1801	-	0.0526	0.1899	-	0.0151	-	-	0.5708	-	-	-	-
*NPQ*	0.7772	0.0009	0.0511	-	0.4098	0.8606	-	0.2819	-	-	0.3618	-	-	-	-
*Ψ_l_*	0.0060	0.0033	0.2741	<0.0001	0.0081	0.6432	<0.0001	0.7893	<0.0001	0.1423	0.9707	<0.0001	0.0642	0.2988	0.0112

- Does not apply.

**Table 3:** Mean ± SE values for the net CO_2_ assimilation rate (*A*) (μmol CO_2_ m^−2^ s^−1^) at light saturation (1295 μmol photons m^−2^ s^−1^ and 90 s illumination time), transpiration rate (*E*) (mmol H_2_O m^−2^ s^−1^), stomatal conductance (*g_s_*) (mmol H_2_O m^−2^ s^−1^), intercellular CO_2_ concentration (*Ci*) (ppm), leaf temperature (*LT*) (°C), extrinsic water use efficiency (*WUEe*) (μmol CO_2_ mmol H_2_O^−1^), intrinsic water use efficiency (*WUEi*) (μmol CO_2_ mmol H_2_O^−1^), actual photochemical efficiency of PSII (*Φ_PSII_*), electron transport rate (*ETR*), maximum photochemical efficiency of PSII (*F_v_/F_m_*), efficiency of excitation energy captured by open PSII reaction centers (*F_v_’/F_m_’*), photochemical quenching coefficient (*qP*), non-photochemical quenching coefficient (*NPQ*) and leaf water potential (*Ψ_L_*) (MPa), for each studied main fixed effect.

Effect	Level	Variables													
		*A*	*E*	*g_s_*	*Ci*	*LT*	*WUEe*	*WUEi*	*Φ_PSII_*	*ETR*	*F_v_/F_m_*	*F_v_´/F_m_´*	*qP*	*NPQ*	*Ψ_L_*
Period	Rainy	12.63 ± 0.20 ^a^	4.36 ± 0.05 ^a^	372.37 ± 8.00 ^a^	304.28 ± 2.01 ^a^	28.09 ± 0.06 ^b^	2.79 ± 0.08 ^b^	0.013 ± 0.0025 ^b^	0.15 ± 0.0032 ^b^	82.64 ± 1.80 ^b^	0.75 ± 0.003 ^b^	0.72 ± 0.004 ^b^	0.92 ± 0.007 ^a^	0.018 ± 0.005	−0.13 ± 0.0033 ^a^
	Dry	10.47 ± 0.22 ^b^	3.27 ± 0.05 ^b^	331.59 ± 8.00 ^b^	290.76 ± 1.66 ^b^	29.53 ± 0.06 ^a^	3.11 ± 0.05 ^a^	0.039 ± 0.0014 ^a^	0.17 ± 0.0030 ^a^	94.33 ± 1.65 ^a^	0.79 ± 0.002^a^	0.75 ± 0.003 ^a^	0.90 ± 0.005 ^b^	0.018 ± 0.001	−0.14 ± 0.0021 ^b^
Site	Semi-humid warm	11.08 ± 0.20 ^b^	3.62 ± 0.06 ^b^	309.28 ± 9.27 ^b^	294.72 ± 2.00 ^b^	28.98 ± 0.07 ^a^	3.05 ± 0.06 ^a^	0.031 ± 0.0023 ^a^	0.15 ± 0.0036 ^b^	82.63 ± 2.01 ^b^	0.76 ± 0.002 ^b^	0.73 ± 0.004 ^b^	0.89 ± 0.01 ^b^	0.016 ± 0.005 ^b^	−0.13 ± 0.0025 ^a^
	Humid warm	12.02 ± 0.20 ^a^	4.01 ± 0.06 ^a^	394.68 ± 9.27 ^a^	300.32 ± 2.00 ^a^	28.64 ± 0.07 ^b^	2.85 ± 0.06 ^b^	0.021 ± 0.0023 ^b^	0.17 ± 0.0036 ^a^	94.34 ± 2.01 ^a^	0.78 ± 0.003 ^a^	0.75 ± 0.005 ^a^	0.94 ± 0.01 ^a^	0.021 ± 0.001^a^	−0.14 ± 0.0025 ^b^
Genotype	ECC 25	10.71 ± 0.44 ^d^	3.20 ± 0.13 ^c^	302.00 ± 19.30 ^b^	290.52 ± 3.48 ^b^	28.72 ± 0.09 ^b^	3.29 ± 0.14 ^a^	0.027 ± 0.0038	0.15 ± 0.01	82.59 ± 3.42	0.77 ± 0.009 ^a,b,c^	0.74 ± 0.007	0.92 ± 0.01	0.020 ± 0.001	−0.14 ± 0.0052
	ECC 29	10.95 ± 0.44 ^c^	3.76 ± 0.13 ^b^	303.92 ± 19.30 ^b^	300.37 ± 3.48 ^a^	28.94 ± 0.09 ^a^	2.68 ± 0.14 ^b^	0.027 ± 0.0038	0.16 ± 0.01	87.99 ± 3.42	0.77 ± 0.009 ^a,b,c^	0.74 ± 0.009	0.93 ± 0.01	0.015 ± 0.001	−0.13 ± 0.0052
	ECC 35	11.17 ± 0.44 ^bd^	3.45 ± 0.13 ^c^	317.30 ± 19.30 ^b^	294.21 ± 3.48 ^b^	28.49 ± 0.09 ^b^	3.02 ± 0.14 ^b^	0.024 ± 0.0038	0.16 ± 0.01	88.37 ± 3.42	0.75 ± 0.003 ^d^	0.73 ± 0.004	0.89 ± 0.01	0.021 ± 0.002	−0.13 ± 0.0052
	ECC 60	11.78 ± 0.44 ^ad^	4.25 ± 0.13 ^a^	386.77 ± 19.30 ^a^	305.70 ± 3.48 ^a^	29.09 ± 0.09 ^a^	2.71 ± 0.14 ^b^	0.025 ± 0.0038	0.16 ± 0.01	88.15 ± 3.42	0.77 ± 0.003 ^a,b,c^	0.73 ± 0.007	0.90 ± 0.01	0.018 ± 0.001	−0.14 ± 0.0052
	ECC 64	10.65 ± 0.44 ^d^	3.77 ± 0.13 ^b^	330.78 ± 19.30 ^b^	298.74 ± 3.48 ^a^	29.03 ± 0.09 ^a^	2.89 ± 0.14 ^b^	0.030 ± 0.0038	0.15 ± 0.01	81.99 ± 3.42	0.77 ± 0.003 ^b,c,d^	0.74 ± 0.005	0.91 ± 0.02	0.017 ± 0.001	−0.14 ± 0.0052
	ECC 66	11.45 ± 0.44 ^bd^	3.99 ± 0.13 ^a^	348.01 ± 19.30 ^b^	302.93 ± 3.48 ^a^	28.92 ± 0.09 ^a^	2.64 ± 0.14 ^b^	0.026 ± 0.0038	0.16 ± 0.01	87.57 ± 3.42	0.76 ± 0.004 ^b,c,d^	0.74 ± 0.008	0.92 ± 0.01	0.016 ± 0.001	−0.13 ± 0.0052
	ECC 73	12.20 ± 0.44 ^a,b^	3.66 ± 0.13 ^b^	342.09 ± 19.30 ^b^	286.78 ± 3.48 ^b^	29.06 ± 0.09 ^a^	3.32 ± 0.14 ^a^	0.028 ± 0.0038	0.18 ± 0.01	94.65 ± 3.42	0.78 ± 0.004 ^a^	0.74 ± 0.006	0.93 ± 0.01	0.020 ± 0.001	−0.14 ± 0.0052
	ECC 83	12.01 ± 0.44 ^ac^	3.78 ± 0.13 ^b^	368.37 ± 19.30 ^a^	292.55 ± 3.48 ^b^	28.71 ± 0.09 ^b^	3.31 ± 0.14 ^a^	0.027 ± 0.0038	0.17 ± 0.01	89.52 ± 3.42	0.77 ± 0.003 ^a,b^	0.74 ± 0.005	0.92 ± 0.01	0.018 ± 0.001	−0.13 ± 0.0052
	ECC 90	12.76 ± 0.44 ^a^	4.12 ± 0.13 ^a^	431.48 ± 19.30 ^a^	298.90 ± 3.48 ^a^	28.50 ± 0.09 ^b^	2.93 ± 0.14 ^b^	0.022 ± 0.0038	0.18 ± 0.01	96.24 ± 3.42	0.78 ± 0.003 ^b,c,d^	0.74 ± 0.006	0.91 ± 0.01	0.018 ± 0.001	−0.14 ± 0.0052
	IAN 873	11.81 ± 0.44 ^ad^	4.18 ± 0.13 ^a^	389.06 ± 19.30 ^a^	304.50 ± 3.48 ^a^	28.65 ± 0.09 ^b^	2.69 ± 0.14 ^b^	0.024 ± 0.0038	0.16 ± 0.01	87.80 ± 3.42	0.76 ± 0.003 ^c,d^	0.75 ± 0.006	0.90 ± 0.01	0.019 ± 0.001	−0.13 ± 0.0052
Hour	3	-	-	-	-	-	-	-	-	-	-	-	-	-	−0.08 ± 0.0015 ^a^
	6	9.02 ± 0.30 ^d^	3.81 ± 0.07 ^c^	323.17 ± 11.24 ^d^	309.52 ± 3.53 ^b^	28.06 ± 0.05 ^d^	2.49 ± 0.14 ^c^	0.021 ± 0.0015 ^b^	0.12 ± 0.0039 ^c^	65.84 ± 2.15 ^c^	-	-	-	-	−0.11 ± 0.0038 ^b^
	9	16.84 ± 0.30 ^a^	4.72 ± 0.07 ^a^	500.75 ± 11.24 ^a^	283.76 ± 3.90 ^c^	28.67 ± 0.14 ^c^	3.65 ± 0.15 ^b^	0.024 ± 0.0024 ^b^	0.22 ± 0.0036 ^a^	119.25 ± 1.97 ^a^	-	-	-	-	−0.16 ± 0.0035 ^c^
	12	16.08 ± 0.26 ^b^	4.42 ± 0.07 ^b^	420.35 ± 11.24 ^b^	269.62 ± 1.98 ^d^	29.76 ± 0.14 ^a^	3.85 ± 0.11 ^a^	0.033 ± 0.0018 ^a^	0.22 ± 0.0035 ^a^	120.99 ± 1.92 ^a^	-	-	-	-	−0.18 ± 0.0036 ^d^
	15	13.64 ± 0.23 ^c^	4.00 ± 0.07 ^c^	377.68 ± 11.24 ^c^	273.44 ± 3.00 ^d^	29.06 ± 0.11 ^b^	3.92 ± 0.11 ^a^	0.036 ± 0.0019 ^a^	0.19 ± 0.0035 ^b^	102.89 ± 1.95 ^b^	-	-	-	-	−0.16 ± 0.0036 ^c^
	18	2.17 ± 0.32 e	2.12 ± 0.07 ^d^	137.95 ± 11.24 e	351.25 ± 3.22 ^a^	28.49 ± 0.07 ^c^	0.83 ± 0.12 ^d^	0.017 ± 0.0019 ^c^	0.06 ± 0.0043 ^d^	33.46 ± 2.34 ^d^	-	-	-	-	−0.12 ± 0.0037 ^b^

Values in columns followed by the same letter or without letter do not differ statistically (Fisher’s least significant difference LSD test, *p* < 0.05).

**Table 4:** Girth (cm) of nine *Hevea brasiliensis* genotypes from the ECC-1 (Élite Caquetá Colombia) selection and IAN 873 cultivar (control) after one year of growth (i.e., 3-year-old trees) at two sites with different climates in Caquetá (Northwestern Colombian Amazon).

Genotype	Humid Warm	Semi-Humid Warm	Average
ECC 25	21.30 ± 0.62 ^b,c,d^	21.37 ± 0.71 ^b,c,d^	21.33 ^b,c^
ECC 29	23.18 ± 0.81 ^a,b^	20.88 ± 0.82 ^d^	22.03 ^a,b^
ECC 35	21.26 ± 0.65 ^b,c,d^	21.25 ± 0.57 ^b,c,d^	21.25 ^b,c^
ECC 60	21.54 ± 0.78 ^b,c,d^	20.78 ± 0.75 ^c,d^	21.16 ^b,c^
ECC 64	23.36 ± 0.52 ^a^	20.82 ± 0.78 ^b,c,d^	22.09 ^a,b^
ECC 66	21.14 ± 0.78 ^b,c,d^	21.43 ± 0.47 ^b,c,d^	21.28 ^b,c^
ECC 73	23.17 ± 0.51 ^a,b^	22.66 ± 0.56 ^a,b^	22.91 ^a,b^
ECC 83	23.16 ± 0.32 ^a,b^	22.86 ± 0.36 ^a,b^	23.01 ^a^
ECC 90	23.14 ± 0.48 ^a,b^	22.96 ± 0.96 ^a,b^	23.05 ^a^
IAN 873	20.72 ± 0.88 ^c,d^	20.25 ± 0.86 ^c,d^	20.49 ^c^
Mean	22.20 ^a^	21.53 ^a^	

Values represent the mean ± SE of four replications (*n* = 4). Means followed by the same letter do not differ statistically (Fisher’s LSD test, *p* < 0.05).

## Data Availability

Data are available from the authors upon request.

## Supplementary Materials

The following are available online at https://www.mdpi.com/article/10.3390/plants10112320/s1, Table S1: Mean and standard error values for the soil volumetric water content (*VWC*), soil water potential (*Ψ_s_*) and soil temperature (*ST*).
