# Supplementary material for: Photosynthesis-Related Responses of Colombian Elite Hevea brasiliensis Genotypes under Different Environmental Variations: Implications for New Germplasm Selection in the Amazon
Source: Plants (Basel). 2021 Oct 28;10(11):2320. doi: 10.3390/plants10112320 (PMC8620879; doi:10.3390/plants10112320)
Supplement: Supplementary file 1 [file plants-10-02320-s001.zip › plants-1409497-supplementary.pdf]

Supplementary Materials

Photosynthesis-related responses of Colombian elite *Hevea brasiliensis* genotypes under different environmental variations: implications for new germplasm selection in the Amazon

Armando Sterling[1,*], Lised Guaca-Cruz[1,2], Edwin Andrés Clavijo-Arias[1], Natalia Rodríguez-Castillo[1] and Juan Carlos Suárez[3]

1 Laboratorio de Fitopatología, Instituto Amazónico de Investigaciones Científicas Sinchi – Facultad de Ciencias Básicas - Universidad de la Amazonía, Florencia, Caquetá, 180001, Colombia; lisguacacruz@gmail.com (L.G.-C); andresclavijoarias@gmail.com (E.A.C.-A.); narodriguezc16@gmail.com (N.R.-C.)

2 Programa Doctorado en Ciencias Naturales y Desarrollo Sustentable, Facultad de Ciencias Agropecuarias, Universidad de la Amazonía, Florencia, Caquetá, Colombia

3 Laboratorio de Ecofisiología, Universidad de la Amazonia, Facultad de Ingeniería, Programa de Ingeniería Agroecológica. Florencia, Caquetá, 180001, Colombia; ju.suarez@udla.edu.co (J.C.S.)

* Correspondence: asterling@sinchi.org.co (A.S.); Tel.: +57-3107862496 (A.S.)

Supplementary Tables

Table S1. Mean and standard error values for the soil volumetric water content (*VWC*), soil water potential (Ψ_s) and soil temperature (*ST*).

Site	Period	Hour	*VWC* (%)	*Ψs* (MPa)	*ST* (°C)
Humid warm	Rainy	3:00	38.66 ± 0.58	-0.009 ± 0.0002	27.48 ± 0.06
		6:00	38.29 ± 0.58	-0.010 ± 0.0002	27.47 ± 0.06
		9:00	40.02 ± 0.60	-0.011 ± 0.0002	27.41 ± 0.05
		12:00	39.20 ± 0.60	-0.011 ± 0.0002	27.39 ± 0.06
		15:00	37.23 ± 0.50	-0.010 ± 0.0011	27.83 ± 0.10
		18:00	39.04 ± 0.94	-0.010 ± 0.0002	28.26 ± 0.12
	Dry	3:00	39.40 ± 0.56	-0.010 ± 0.0067	28.52 ± 0.16
		6:00	38.63 ± 0.61	-0.011 ± 0.0067	28.09 ± 0.23
		9:00	39.18 ± 0.60	-0.012 ± 0.0002	27.51 ± 0.18
		12:00	36.10 ± 0.48	-0.020 ± 0.0002	27.59 ± 0.11
		15:00	37.40 ± 0.54	-0.011 ± 0.0002	27.85 ± 0.09
		18:00	36.50 ± 0.50	-0.010 ± 0.0002	28.16 ± 0.09
Semi-humid warm	Rainy	3:00	37.84 ± 0.61	-0.010 ± 0.0002	27.07 ± 0.12
		6:00	39.12 ± 0.52	-0.010 ± 0.0002	26.93 ± 0.10
		9:00	39.42 ± 0.40	-0.011 ± 0.0002	26.53 ± 0.09
		12:00	37.08 ± 0.57	-0.011 ± 0.0002	26.52 ± 0.09
		15:00	38.67 ± 0.44	-0.010 ± 0.0002	26.90 ± 0.09
		18:00	38.45 ± 0.41	-0.010 ± 0.0002	26.89 ± 0.08
	Dry	3:00	35.58 ± 0.65	-0.028 ± 0.0028	27.04 ± 0.54
		6:00	35.53 ± 0.63	-0.032 ± 0.0027	27.11 ± 0.11
		9:00	35.35 ± 0.67	-0.034 ± 0.0025	27.81 ± 0.09
		12:00	34.05 ± 0.67	-0.036 ± 0.0024	28.17 ± 0.07
		15:00	35.36 ± 0.72	-0.035 ± 0.0026	27.14 ± 0.10
		18:00	35.37 ± 0.73	-0.033 ± 0.0027	27.08 ± 0.13
